# Interventions to improve antiretroviral therapy adherence among adolescents in low- and middle-income countries: A systematic review of the literature

**DOI:** 10.1371/journal.pone.0189770

**Published:** 2018-01-02

**Authors:** Kathleen Ridgeway, Lisa S. Dulli, Kate R. Murray, Hannah Silverstein, Leila Dal Santo, Patrick Olsen, Danielle Darrow de Mora, Donna R. McCarraher

**Affiliations:** 1 Global Health, Population, & Nutrition, Durham, NC, United States of America; 2 Maternal and Child Health, Gillings School of Global Public Health, University of North Carolina at Chapel Hill, NC, United States of America; 3 Global Health, Population, & Nutrition, Washington, D.C., United States of America; National and Kapodistrian University of Athens, GREECE

## Abstract

**Introduction:**

Globally, an estimated 30% of new HIV infections occur among adolescents (15–24 years), most of whom reside in sub-Saharan Africa. Moreover, HIV-related mortality increased by 50% between 2005 and 2012 for adolescents 10–19 years while it decreased by 30% for all other age groups. Efforts to achieve and maintain optimal adherence to antiretroviral therapy are essential to ensuring viral suppression, good long-term health outcomes, and survival for young people. Evidence-based strategies to improve adherence among adolescents living with HIV are therefore a critical part of the response to the epidemic.

**Methods:**

We conducted a systematic review of the peer-reviewed and grey literature published between 2010 and 2015 to identify interventions designed to improve antiretroviral adherence among adults and adolescents in low- and middle-income countries. We systematically searched PubMed, Web of Science, Popline, the AIDSFree Resource Library, and the USAID Development Experience Clearinghouse to identify relevant publications and used the NIH NHLBI Quality Assessment Tools to assess the quality and risk of bias of each study.

**Results and discussion:**

We identified 52 peer-reviewed journal articles describing 51 distinct interventions out of a total of 13,429 potentially relevant publications. Forty-three interventions were conducted among adults, six included adults and adolescents, and two were conducted among adolescents only. All studies were conducted in low- and middle-income countries, most of these (n = 32) in sub-Saharan Africa. Individual or group adherence counseling (n = 12), mobile health (mHealth) interventions (n = 13), and community- and home-based care (n = 12) were the most common types of interventions reported. Methodological challenges plagued many studies, limiting the strength of the available evidence. However, task shifting, community-based adherence support, mHealth platforms, and group adherence counseling emerged as strategies used in adult populations that show promise for adaptation and testing among adolescents.

**Conclusions:**

Despite the sizeable body of evidence for adults, few studies were high quality and no single intervention strategy stood out as definitively warranting adaptation for adolescents. Among adolescents, current evidence is both sparse and lacking in its quality. These findings highlight a pressing need to develop and test targeted intervention strategies to improve adherence among this high-priority population.

## Introduction

Recent years have seen great improvements in access to antiretroviral therapy (ART) for people living with HIV, as global ART coverage has more than doubled from 2010 to 2015 [1]. These efforts, however, are insufficient to ensure positive health outcomes; patients must be highly adherent to ART regimens in order to achieve viral suppression [2, 3] and experience reduced likelihood of HIV-related mortality [4, 5], drug resistance [6–8], and secondary HIV transmission [9]. Taking at least 95% of all ART doses is widely regarded as a standard benchmark for adequate adherence [3].

Global stakeholders and decision-makers have recently prioritized targeted programming and differentiated care for adolescents with HIV in response to a growing burden among young people [10–13]. Thirty percent of new HIV infections occurred among adolescents (15–24 years) in 2014, and HIV is the second leading cause of death among adolescents globally [14–16]. The burden of the epidemic lies largely in sub-Saharan Africa, where the prevalence is estimated to be 2.2% among young women (15–25 years) and 1.1% among young men compared to global estimates of 0.4% and 0.3%, respectively [17].

Estimates of ART adherence among adolescents living with HIV (ALHIV) in low- and middle-income countries (LMIC) vary substantially. A 2014 systematic review found estimates of adherence ranging from 16% to 99% among adolescent populations globally [18]; a meta-analysis of data for adolescents and young adults (12–24 years) in 53 countries, also from 2014, found adherence based on either self-report or viral load measures was 84% in both Africa and Asia [19]. Virologic data for ALHIV are limited but indicate that rates of viral suppression (<400 copies/mL) range from 27% to 89% in Africa, from 52% to 87% in Asia, and from 37.5% to 49% in Central and South America [20]. Expanding HIV testing efforts and new “Test and Treat” or “Test and Start” programming in many LMIC will see a larger number of patients diagnosed with HIV and immediately eligible for treatment, challenging providers to ensure high adherence among a larger, likely healthier patient population [21].

Given the limited body of evidence on adherence interventions for ALHIV [22–24], this systematic review included interventions designed to increase ART adherence among both HIV-infected adults and adolescents in LMIC. The specific objectives of this review were to 1) identify interventions available in the peer-reviewed and grey literature designed to increase ART adherence among adults and adolescents, 2) describe the body of literature in both populations, and 3) identify evidence-based intervention strategies that have potential to be scaled-up or adapted for ALHIV in LMIC. Although numerous systematic reviews have evaluated the effectiveness of adherence interventions for the general population [25–34], few have focused specifically on ALHIV in LMIC and none to date have included literature from adult populations to propose ALHIV-specific recommendations.

## Methods

We used a systematic search strategy to search PubMed, Web of Science, Popline, the USAID Development Experience Clearinghouse [35], and USAID’s AIDSFree Project website [36] (see supporting information). Teams of reviewers (among KR, KM, LD, DM, PO, DD, and LCD) conducted title, abstract, and full text review and evaluated the methodological quality of each publication with the aid of the NIH National Heart, Lung, and Blood Institute (NHLBI) quality assessment tools [37]. Additional methodological details are available in a companion article, which reviews interventions designed to increase retention of adults and adolescents in HIV care [38].

A study was eligible to be included if it met all of the following criteria: 1) evaluated the effects of or examined the associations between an intervention or program and ART adherence or retention in HIV care; 2) reported quantitative measures of ART adherence or retention in care; 3) conducted among adults (age ≥18) or adolescents (mean age 10–19); and 4) published within the five-year search period (20 November 2010 to 20 November 2015). Letters, editorials, conference abstracts, and presentations were not eligible for inclusion. We excluded studies that were not available in English or were conducted in World Bank high-income countries [39]. Pre-exposure prophylaxis (PrEP) and post-exposure prophylaxis (PEP) interventions, interventions to increase HIV testing rates or increase engagement in pre-ART care, and pharmaceutical interventions to change drug combinations were excluded. We also excluded interventions tailored to the specific needs of sub-populations (e.g. prisoners, people who inject drugs). This article focuses on interventions to improve ART adherence; a companion article describes interventions to improve retention in HIV care [38].

For this review, we included studies that reported any quantitative measure of ART adherence including viral load, measures of drug concentrations, CD4 cell counts, self-reported adherence using either novel or validated measures, pill counts, and data from electronic adherence monitoring devices (EAMDs).

## Results and discussion

**Fig 1:**
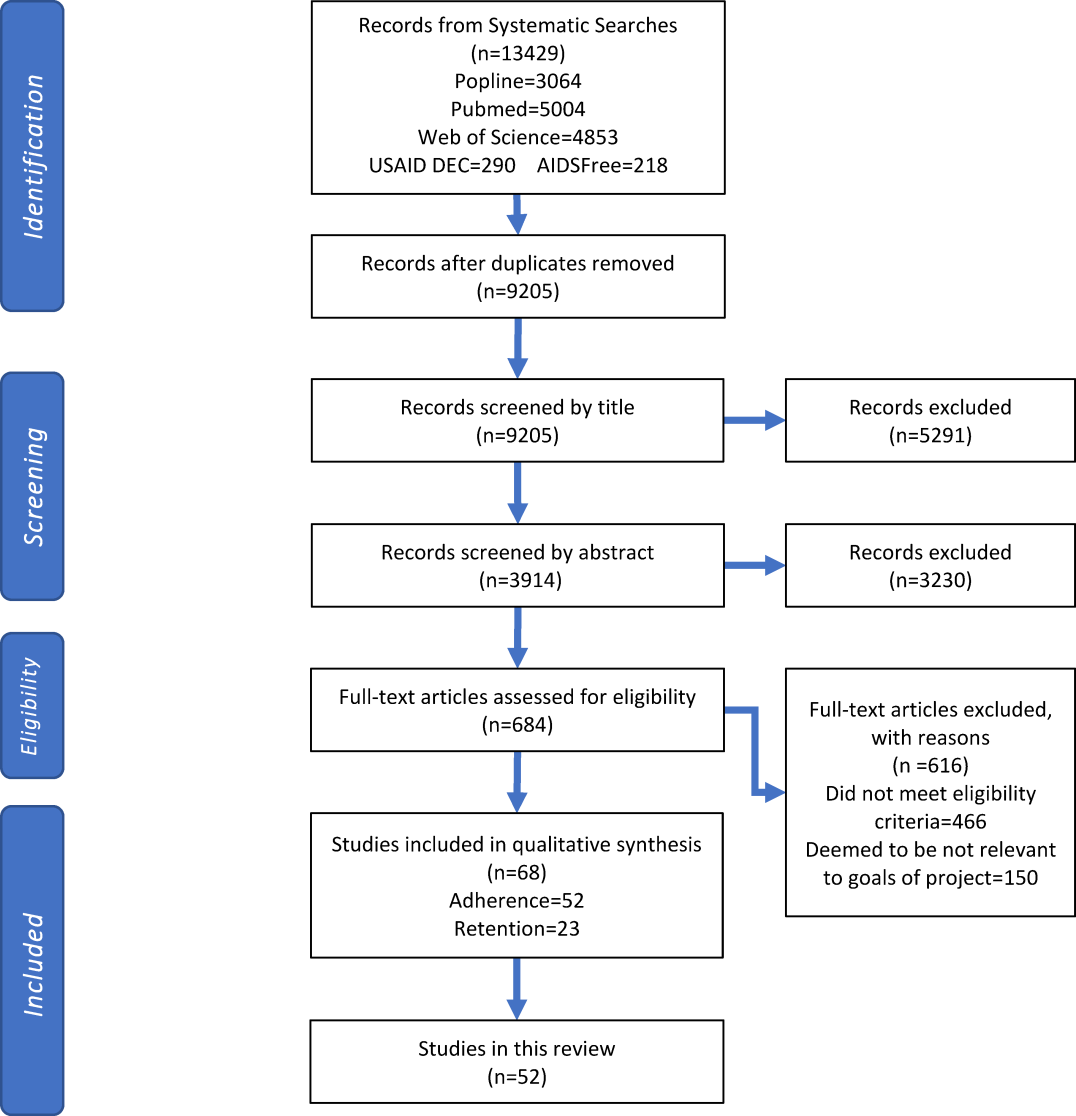
Selection process for inclusion of studies.

We identified 13,429 potentially relevant publications, of which fifty-two were eligible for inclusion in this review (Fig 1). Forty-five studies were conducted among adults, five were conducted among both adults and adolescents, and two were conducted among adolescents only. Fourteen of the adult studies were of good methodological quality, 15 of fair quality, and 16 of poor quality. Among the five studies that included both adults and adolescents, one was rated good, one was rated fair, and three were rated poor quality. Both of the studies that involved adolescents exclusively were fair quality.

All 52 studies were published in peer-reviewed journals. We found 33 single-country studies from sub-Saharan Africa (10 countries), 11 from Asia (four countries), five from Central and South America (three countries), one from Haiti, and one from Pakistan. There was one multi-country study that was conducted in five sub-Saharan African countries, Brazil, Haiti, and Peru.

The 52 publications described 51 unique interventions. Among studies targeting adults exclusively, mHealth-based interventions (n = 12), community- or home-based care (n = 10), and individual or group adherence counseling (n = 8) were most common (Table 1). Other interventions among adults included instrumental support (n = 5), task shifting or decentralization (n = 4), multi-component facility-based interventions (n = 3), pharmacist counseling (n = 1), and depression treatment (n = 1). Interventions for combined adult and adolescent study populations included adherence counseling (n = 2), community-based adherence support (n = 2), and short message service (SMS) reminders (n = 1). Both studies conducted with adolescents evaluated group adherence counseling interventions.

**Table 1:** Description of intervention types.

Intervention Type	Definition	Adolescents (10–19)	Adults + Adolescents	Adults Only (18+)	Total
**Adherence Counseling**					
Individual counseling	Adherence educational and/or counseling interventions delivered in a one-on-one setting. Sessions are often led by trained health professionals or lay counsellors.	0	1	3	**4**
Group counseling	Adherence educational and/or counseling interventions delivered in a group setting. Includes social support groups. Sessions are often led by trained professionals or lay counsellors and delivered through a set curriculum or informed by a psychosocial theory/practice.	2	0	5	**7**
Individual plus group counseling	Interventions with both individual and group counseling components.	0	1	0	**1**
**mHealth Interventions**					
SMS reminders sent at regular intervals	Regular delivery of SMS messages aimed at directly or indirectly reminding patients to adhere to their medication. Many interventions did not mention HIV or ART in the messages and several utilized the text as a way to check in with the patient.	0	0	4	**4**
SMS reminders triggered by adherence monitors	SMS messages were sent if an electronic adherence monitoring device were not opened within 30 minutes of the schedule dose time.	0	1	1	**2**
IVR or phone calls for reminders	Interactive voice response or regular phone calls delivering messages on medication adherence and other HIV/ART related topics, as well as appointment reminders. Some also sent non-interactive, SMS picture messages to remind patients of dosage adherence.	0	0	4	**4**
SMS or alarm reminders plus individual counseling	Individual adherence counseling combined with regular reminders. Reminders could come in the form of either an alarm device programmed around dosage times or regular SMS messages, sent at times independent of the dosage schedule.	0	0	3	**3**
**Community- and Home-based strategies**					
CBAS with home visits	Provision of adherence support through home visits by a community-based worker or volunteer. Home visitors can range from peer educators to community health workers. They are involved in a variety of activity such as DOT, basic clinical assessments and patient referrals, pill counts, food ration provision, and ART delivery.	0	2	6	**8**
Multi-component facility- and community-based program	Interventions involving a facility-designated worker who connects patients to facility-level services. Responsibilities of the worker have included home visits, patient monitoring and tracing, and counseling.	0	0	1	**1**
Peer treatment supporters	Involving other persons within a patient's social circle in assisting that patient with their treatment. Examples of responsibilities of the treatment supporter include performing DOT, positive social support, clinical site mediation, follow-up appointment attendance.	0	0	2	**2**
Community-based social network support	Support at the community level designed to be delivered to a group. Includes interventions involving a patient's extended social network in that patient's treatment.	0	0	1	**1**
**Pharmacist Counseling**					
Pharmacist counseling	Shifting patient counseling to occur when patients receive their medication. Counseling was provided by pharmacist and drug-related problems were addressed at each scheduled meeting.	0	0	1	**1**
**Depression Treatment**					
Depression treatment	Integrating ART and mental health care, with the overall goal of addressing the underlying depression-related factors related to adherence.	0	0	1	**1**
**Facility-based Interventions**					
Facility-based interventions	Multi-component interventions that are delivered and/or organized at the facility level. Examples of activities include individual and group counseling, patient-fast tracking, educational classes and materials. These interventions tend to focus on providing comprehensive support and care to ART patients.	0	0	3	**3**
**Instrumental Support**					
Nutrition support	Providing supplementary nutritional support either at the individual or household level. Can come in the form of food rations or nutrition education.	0	0	4	**4**
Disability grants	Providing monetary grants to people that meet clinical criteria for advanced stages of HIV to provide support until they are well enough to return to work.	0	0	1	**1**
**Decentralization, Down-referral, and Task-shifting**					
Task-shifting	Refers to transferring specific responsibilities for HIV treatment and care from physicians toward other health care workers such as clinical officers and nurses.	0	0	3	**3**
Decentralization	Shifting service delivery for stable patients to a lower-level of care. Decentralization occurs at the facility level, such as transitioning care from hospital-based to clinic-based care. Down-referral usually denotes changes in the provider level, like changing primary ART provision from hospital-based to general-practitioner based.	0	0	1	**1**
	**Total**	**2**	**5**	**44**	**51**

Note: 51 interventions were described in 52 publications. Two publications (Achieng et al., 2012 and Achieng et al., 2013) described the same multi-component facility- and community-based program.

Adherence was measured using varied methods. Biological measures included CD4 counts, ART concentrations in hair samples, and viral load. Viral load measures included viral suppression using cut-offs of <50, <100, <200, or <400 copies/mL; and virologic failure with cut-offs of >40, >400 or >5,000 copies/mL. Pill counts were conducted by clinic staff or lay health workers and measured the number of pills remaining compared to the number of doses that should have been taken over a specified time period. Similarly, EAMDs such as MEMSCap^TM^ or Wisepill^TM^ measured whether and when patients opened pill bottles for each medication. Self-reported measures included the Adult AIDS Clinical Trial Group (AACTG) self-report measure [42], the Antiretroviral General Adherence Scale (AGAS) [43], the Pediatric AIDS Clinical Trials Group (PACTG) self-report measure [44, 45], and Visual Analog Scales (VAS) [46].

The 52 studies presented in this review included randomized controlled trials (RCTs) (n = 29), quasi-experimental studies (n = 5), single-group pre-test/post-test studies (n = 5), prospective cohort studies (n = 6), and retrospective cohort studies (n = 4). Studies are grouped by type and discussed below, with detailed information in Table 2.

**Table 2:** Characteristics of included studies.

Author, year	Study Year	Country	Population and Setting	Study Design	Intervention Description	Outcomes	Results
***Adherence counseling***							
Basso et al., 2013 [47]	2008	Brazil	HIV-infected adults with VL >50 copies/ml, on ART ≥6 months.	RCT	Individual adherence counseling sessions conducted by previously trained health professionals, held in four 1-hour sessions every 15 days. Session content identified obstacles to treatment adherence, increased knowledge about their treatment, answered questions about ART, and identified ways to face difficulties with adhering to ART.	% doses taken (EAMD: MEMSCap)	No statistically significant differences in mean % of doses taken between intervention and control groups at any time point (30, 60, 90, and 120 days). No test statistics were reported.
			N = 121 (64 intervention, 57 control)			% doses taken at prescribed time (EAMD: MEMSCap)	No statistically significant differences in mean % of doses taken at prescribed time between intervention and control groups at any time point (30, 60, 90 and 120 days). No test statistics were reported.
					Control: Usual care consisting of consultations with physicians every 2 months, or more frequently when clinically indicated.	VL	No statistically significant difference in final log VL between the intervention (n = 44) and control (n = 36) groups. No test statistics were reported.
Robbins et al, 2015 [48]	2008–2010	South Africa	HIV-infected adults on ART for ≥6 months identified as non-adherent who were willing to bring a treatment support partner to counseling at a public primary health clinic. Non-adherence defined as < 90% adherent by clinic-based pill count, detectable VL, or other clinical sign of non-adherence.	RCT	Multimedia-based, computer-driven intervention led by lay counsellors held in 6 weekly sessions. Participants interacted with the computer platform to answer questions and complete activities. Content included information on HIV, ART, and adherence; problem solving to overcome adherence barriers; videos to highlight importance of treatment support; and treatment supporter participation in counseling sessions.	% doses taken over past 30 days (Pill count: Pharmacy refill data)	No statistically significant difference in mean change of % doses taken over 5–6 week intervention period between the intervention and control groups (β = 0.18, p = 0.17).
						Frequency of taking all doses as prescribed (Self-report: AACTG) [42]	No statistically significant difference in mean change of frequency of taking all doses as prescribed over 5–6 week intervention period between the intervention and control groups (β = -0.01, p = 0.97).
					Control: Standard-of-care adherence counseling with existing staff counsellors at counsellors’ discretion, often in a single, <15-minute session.		*Note*: *pill count data were only available for 10 participants per intervention arm (30*.*8% of participants)*
		
			N = 65 (33 intervention, 32 control)				
Khachani et al., 2012[49]	2006–2007	Morocco	HIV-infected adults on ART ≥2 months and regularly attending consultations at a university hospital.	Single-group pre-test post-test study	Psychoeducational program delivered personalized educational and psychological support sessions lasting 3–5 hours at each medical consultation. Sessions were delivered by a multidisciplinary team including a medical intern, a physician, an educator, and a psychologist. Content included information on HIV transmission, prevention, and HAART; treatment planning; card games; and cognitive and behavioral support.	Adherence score (Proportion of doses taken over past 4 days) (Self-report: AACTG) [42]	No statistically significant change in median adherence scores from baseline to 6 months (p = 0.266); median score = 1.0 (perfect adherence) at all time points.
			N = 50				

Jobanputra et al., 2015 [50]	2012–2013	Swaziland	HIV-infected adults, adolescents, and children who underwent VL testing (n = 12063). 10% of the patient population was < 20 years, median age not reported.	Retrospective cohort study	Enhanced adherence counseling (EAC) delivered by lay counsellors for 3 months.	Viral re-suppression (achieving < 100 copies/mL or 2 log reduction in VL after detectable VL)	Odds of viral re-suppression were not statistically significantly different between the EAC group and the comparison (OR = 0.5 [0.2, 1.1]) after 4 months.
					Comparison: Those with detectable viral load who did not receive EACs services.		
			For counseling sub-analysis, patients with non-suppressed VL who underwent follow-up VL test and had a counseling data documented in their chart (n = 180).				
Holstad et al., 2012 [51]	2008	Nigeria	HIV-infected adult women on ART at an HIV clinic.	Quasi-experimental two-group post-test only study	Group motivational interviewing (MI) intervention delivered by trained facilitators in eight 1.5–2 hour weekly sessions with 7–8 members per group. Content drew from Social Cognitive Theory constructs, followed a structured format, and included discussions of challenges taking ART, reaching ART adherence goals, building self-efficacy, and education on sexual and reproductive health topics.	100% adherence (Self-report: VAS)	A statistically significantly higher proportion of patients took 100% of medications in the intervention group compared to the control group 6 months post-intervention (test statistic not reported, p<0.001).
			Pregnant women were not excluded from the study.			Continuous adherence (Self-report: AGAS) [43]	Mean adherence was statistically significantly higher in the intervention group (mean adherence 93.2%) compared to the control group (mean adherence 77.8%) 6 months post-intervention (Z = -3.581, p<0.001).
			N = 48 (28 intervention, 20 control)			Frequency of missing doses (Self-report: AGAS) [43]	Mean missed dose frequency was statistically significantly lower in the intervention group compared to the control group 6 months post-intervention, with higher scores reflecting better adherence (Z = -3.072, p = 0.002).
					Control: Equivalent group counseling intervention focusing on nutrition, exercise, women's health, and stress reduction.	Never missing a dose (Self-report: AACTG) [42]	A statistically significantly higher proportion of patients in the intervention group reported never missing a dose (92.9% never missed dose) compared to the control group (40.0%) 6 months post-intervention (χ2 = 15.78, df = 1, p<0.0001).
Jones, D. L., Zulu, I. et al, 2013 [52]	2006–2008	Zambia	HIV-infected adults on ART for < 24 months receiving care from a hospital immunology clinic.	RCT	Theory-driven group counseling intervention delivered to groups of 10 participants in 3 monthly sessions. Content shaped by the Information-Motivation-Behavioral Skills (IMB) Model [53] and delivered by trained health staff to: provide information on treatment and adherence; foster treatment motivation and engagement; and build adherence skills by encouraging patient-provider communication, side effect coping, and pill reminder use.	Proportion of participants with no missed doses in past 3 months (Self-report)	Post-intervention, a statistically significantly higher proportion of intervention group participants reported having no missed doses compared to control group participants (χ2 = 4.3, p = 0.004). This difference did not persist after crossover (χ2 = 0.01, p = 0.91).
			N = 160 (77 intervention, 83 control)				
Peltzer et al., 2012 [54]	NR	South Africa	HIV-infected adults on ART for 6–24 months who missed at least 1 dose of ART in past month receiving care at an ART clinic in a hospital.	RCT	Group medication adherence intervention (MAI) delivered by lay health workers in three 1-hour sessions on a monthly basis. Intervention content based on the Health Belief Model [55], included information on HIV, ART, adherence, and medication resistance, and utilized discussion and problem-solving.	100% adherence (No missed doses) over past 4 days (Self-report: AACTG) [42]	No statistically significant difference in change in proportion of patients adherent from pre- to post-intervention between intervention and control group at 3 month follow-up (F = 0.917, p = 0.341).
							No statistically significant difference in improvement in mean CD4 count from pre to post-intervention between intervention and control group at 3 month follow-up (F = 0.675, p = 0.412).
			N = 152 (76 intervention, 76 control)		Control: Standard of care including monthly visits with medical practitioner.	CD4 count	
Cook et al., 2014 [56]	2009–2010	India	HIV-infected adults new to ART receiving care at an immunodeficiency clinic and identified as non-adherent by clinic staff.	RCT	Group medication adherence intervention (MAI) delivered in 3 monthly facilitator-led, gender-concordant, group cognitive behavioral counseling sessions focused on increasing motivation and skills related to ART and HIV. Content included information on HIV and ART, medication adherence and related barriers, and HIV-related coping and social support.	Continuous adherence (Pill count and pharmacy refill data)	No statistically significant difference in change in mean adherence after 3 months on intervention between intervention and control groups (β = -0.828 [-1.749, 0.093]).
							No statistically significant differences in mean adherence after 3 months on intervention in either the intervention group (difference = -0.96 [-1.67, -0.23]) or the control group (difference = -0.13 [-0.70, 0.46]).
			N = 80 (34 intervention, 46 control)		Control: Enhanced standard of care including regularly scheduled provider visits plus 3 monthly time-matched sessions where participants were shown HIV-related educational videos.		
Jones, D.L., Sharma, A., et al., 2013 [57]	NR	India	HIV-infected adults who initiated ART 3 to 12 months prior to recruitment receiving care at an immunodeficiency clinic.	RCT	Interactive group medication adherence intervention (MAI) led by master's-level psychologists in 3 monthly sessions following a structured manual. Content included HIV and ART, adherence, HIV-related coping, and social support.	Improved adherence: Change in proportion of doses taken dichotomized into improved or unchanged/ decreased (Pill count: Pharmacy refill data)	No statistically significant difference in proportions of participants with improved adherence in the intervention and control groups 3 months post-intervention (χ^2^ = 0.54, p = 0.46).
			N = 80 (34 intervention, 46 control)		Control: Standard of care plus time-matched sessions of HIV educational videos on healthy living. Received delayed intervention.		
Kaihin et al., 2015 [58]	2011	Thailand	HIV-infected adolescents (15–24 years) identified as<95% adherent by pharmacy records and receiving ART from at community hospitals.	RCT	Group counseling intervention shaped by empowerment theoretical construct [59] delivered in a structured format in eight 2.5 to 3 hour-long sessions over 18 weeks. Content included information and discussion on HIV and ART, developing adherence goals, and encouraging participants to critically evaluate their own ART adherence, take charge of their current situation, and maintain their own sense of power.	>95% adherence (Pill count: Pharmacy refill data)	Statistically significantly higher proportions of patients had >95% adherence in the intervention group compared to the comparison group 8 weeks post-intervention (χ^2^ = 14.723, df = 1, p<0.001).
			N = 46 (23 intervention, 23 control)		Comparison: Patients receiving standard of care (not described) at a comparison community hospital.		
Bhana et al., 2014 [60]	NR	South Africa	HIV-infected pre-adolescents (10–13 years) and their families; pre-adolescents enrolled in care at one of two clinical sites.	RCT	Group counseling intervention informed by the Collaborative HIV Prevention and Adolescent Mental Health Family Program (CHAMP) principles; delivered by lay counsellors and a psychologist to pre-adolescents and family members in biweekly sessions over 3 months. Sessions followed a structured guideline, included a culturally-tailored cartoon storyline, and covered topics including HIV information, disclosure, coping, ART adherence, and social support.	Frequency of missing medication score: Frequency of medications missed over past 6 months (Self-report: adapted from PACTG) [44] [45]	Mean frequency of missing medication score was marginally significantly higher in the intervention group compared to the control groups 2 weeks post-intervention, with higher scores reflecting better adherence (β = 1.527, p = 0.05).
			N = 65 families (33 intervention, 32 control)				
					Control: Standard of care (not described). Received delayed intervention.		
Surilena et al., 2014 [61]	2011–2012	Indonesia	HIV-infected adult (≥17 years) women infected through a spouse or partner attending the outpatient clinics of two hospitals.	RCT	Individual and group counseling sessions based on rational-emotive-behavior-based therapy (REBT) [62], delivered by a psychiatrist in 8 weekly sessions including 6 individual and 2 group sessions. Intervention used a structured, directed, and objective approach to cognitive modification focusing on tangible problems in participants’ lives related to HIV/AIDS.	Continuous adherence (self-report, instrument not reported)	Mean self-reported adherence was similar among intervention and control groups at baseline (74.0% [69.0, 75.3] intervention versus 77.0% [71.8, 80.3] control) and post-intervention (100% [83.3, 96.7] intervention versus 84.0% [77.5, 87.8] control).
						Continuous adherence (pill count)	Mean pill count adherence was 88% at baseline and 100% at post-intervention in the intervention group.
			N = 160 (80 intervention, 80 control)		Control: Not described.	Undetectable VL (VL<50 copies/mL)	Percent of participants with undetectable VL among the intervention group was 92.5% at baseline and 87.5% at post-intervention; undetectable VL was present in 95.0% of control participants at baseline and 93.9% at post-intervention (No statistical testing due to lack of variation and missing values at follow-up).
							*Note*: *Mean self-reported post-intervention adherence for intervention group is not contained in the 95% confidence interval; results should be interpreted with caution*
***mHealth interventions***							
Lester et al., 2010 [63]	2007–2008	Kenya	HIV-infected adults initiating ART in one of three study clinics.	RCT	Weekly SMS messages sent from a clinic nurse inquiring about participants’ status and reminding them of the availability of phone-based support. Participants were required to respond within 48 hours; participants reporting a problem or failing to respond were called by clinicians.	<95% adherence over past 30 days (Self-report)	Risk of <95% adherence at both 6 and 12 month follow-up was statistically significantly lower among intervention group compared to control (RR = 0.81 [0.69, 0.94]).
			Patients were eligible if they had near daily access to a mobile phone.		Control: Standard of care including ART counseling at ART initiation.	Virologic failure (VL>400 copies/mL)	Risk of virologic failure at 12 month follow-up was statistically significantly lower among intervention group compared to the control (RR = 0.85 [0.72, 0.99]).
			N = 538 (273 intervention, 265 control)				
Pop-Eleches et al., 2011 [64]	2007–2008	Kenya	HIV-infected adults initiating ART <3 months prior to enrolment receiving care at a health center.	RCT	Participants received mobile phones and MEMSCap devices, randomized to receive either short or long text messages delivered on a daily or weekly basis for 48 weeks. Messages did not allow responses from participants and did not mention HIV or ART specifically. Short messages served as reminders to take medications and read, “This is your reminder;” long messages provided additional support and read, “This is your reminder. Be strong and courageous, we care about you.”	>90% adherence (EAMD: MEMSCap)	No statistically significant difference in proportions of participants achieving >90% adherence among any intervention arm compared to the control group at endline (test statistics not reported; p = 0.97 daily short messages, p = 0.07 weekly short messages, p = 0.85 daily long messages, p = 0.08 weekly long messages).
			N = 431 (70 short daily reminders, 72 long daily reminders, 73 short weekly reminders, 74 long weekly reminders, 139 control)				A statistically significantly higher proportion of participants receiving weekly messages achieved >90% adherence compared to the control group at endline (p = 0.03); no significant differences in any other combined group compared to control (test statistics not reported; p = 0.92 daily messages, p = 0.47 short messages, p = 0.47 long messages).
					Control: Received mobile phones and MEMSCap devices but no text messages. All participants attended monthly clinical visits.		
Mbuagbaw et al., 2012 [65]	2010	Cameroon	HIV-infected adults (> 21 years) on ART for at least 1 month at a hospital treatment center.	RCT	Standardized mobile phone text messages (SMS) delivered weekly for 6 months. Message content was varied and contemporary (e.g. messages would contain season’s greetings) and contained a motivational component as well as a reminder component to remind patients to take their medication. SMS messages also included a contact number that participants could call if they needed help. HIV and/or ART were not mentioned specifically.	>95% adherence (Self-report: VAS) [46]	Participants in the intervention group were statistically significantly less likely to have > 95% adherence compared to the control group at 3 months (RR = 0.77 [0.63, 0.94]). No statistically significant differences at 6 months (RR = 1.06 [0.89, 1.29]).
						No missed doses (Self-report)	
			Patients were eligible if they owned a mobile phone and could read text messages.		Control: Standard of care including regular ART counseling and home visits determined on a case-by-case basis.	Number of drug refills (Pharmacy refill data)	No statistically significant differences in risk for reporting any missed doses between intervention and control groups at either 3 months (RR = 0.97 [0.85, 1.10]) or 6 months (RR = 1.01 [0.87, 1.16]).
			N = 200 (101 intervention, 99 control)				Mean numbers of drug refills were comparable among intervention and control groups (2.3 intervention, 2.2 control at 3 months; 3.8 intervention 3.7 control at 6 months).
Da Costa et al., 2012 [66]	2009–2010	Brazil	HIV-infected adult women with VL <400 copies/mL for at least 3 months with CD4 count >200 cells/mm^3^ receiving care at an infectious disease center.	RCT	Automatic SMS messages delivered 30 minutes before the required time of the last required medication dose in a day with an automated message of “The UNIFESP informs: take good care of your health.” Responses were not required. Messages were sent every Saturday and Sunday and every other day during the work week for 4 months.	>95% adherence (Self-report)	No statistically significant difference in proportions of participants with >95% self-reported adherence over the study period between intervention and control groups at endline (Z = 1.1663, p = 0.2435).
						>95% adherence (Pill count: Clinic-based)	No statistically significant difference in proportions of participants >95% adherent by pill count over the study period between intervention and control groups at endline (Z = 0.5198, p = 0.6038).
						>95% adherence (EAMD: MEMSCap)	No statistically significant difference in proportions of participants > 95% adherent by EAMD over the study period between intervention and control groups at endline (Z = 1.2972, p = 0.1946).
			Participants were eligible if they owned a mobile phone.		Control: Standard of care including monthly clinical visits.		
			N = 23 (15 intervention, 8 control)				
Sabin et al., 2015 [67]	2012–2013	China	HIV-infected adults receiving or initiating ART at an ART clinic.	RCT	Participants were given an EAMD (Wisepill); participants received EAMD-triggered SMS reminders if Wisespill device was not opened within 30 minutes past the scheduled dose time. Text messages were personalized and did not refer specifically to HIV or ART. Behaviorally-targeted adherence counseling was provided for patients with adherence <95% in the previous month measured by the EAMD. Intervention lasted approximately 6 months.	≥95% adherence (EAMD: Wisepill)	Participants in the intervention group were significantly more likely to have ≥95% adherence at endline compared to the control group (RR = 1.69 [1.29, 2.21]).
			Patients were eligible if they owned a mobile phone and were deemed at risk for poor adherence (new to ART, previous non-adherence, mental health diagnosis, or substance abuse problems).			Continuous adherence (EAMD: Wisepill)	Mean adherence was not statistically significantly different between intervention and control group at baseline (p = 0.970), but was significantly higher among the intervention group at endline (Mean adherence 96.2% intervention, 89.1% control, p = 0.003).
						Undetectable VL (VL <50 copies/mL)	No statistically significant differences in % with undetectable VL at endline between intervention and control groups (p = 0.218).
					Control: Standard of care including monthly clinic visits and targeted adherence counseling provided for patients with self-reported adherence <95%. Wisepill monitor also provided.		
			N = 119 (63 intervention, 56 control)				
Orrell et al., 2015 [68]	2012–2014	South Africa	HIV-infected ART-naïve adults and adolescents (> 15 years) receiving treatment at a treatment center. Mean age 34.5 years, outcomes not stratified by age group.	RCT	Participants received an EAMD (Wisepill) for 48 weeks, which triggered SMS reminders if the Wisespill device was not opened within 30 minutes past the scheduled dose time. Reminder messages were standardized and did not refer specifically to HIV or ART; participants could choose what reminder message they would like to receive. Examples include “Have you forgotten something?” or “Just take it!” Duration of intervention not reported.	Cumulative adherence over study period (EAMD: Wisepill)	No statistically significant differences in cumulative adherence over the study period between intervention and control groups at endline (OR = 1.08 [0.77, 1.52]).
						Virologic failure (VL >40 copies/mL)	No statistically significant difference in the odds of virologic failure between intervention and control groups at endline (OR = 0.77 [0.42, 1.40]).
							*Note*: *Authors reported that adherence was measured as a continuous variable in this analysis but do not provide an interpretation of the odds ratio reported in the results*
			Participants were eligible if they owned their own mobile phone.		Control: Standard of care including monthly, and later bi-monthly clinic visits, pill counts with targeted adherence counseling, and patient tracking for missed visits. Control group also received Wisepill monitor and three pre-treatment education sessions.		
			N = 230 (115 intervention, 115 control)				
Swendeman et al., 2015 [69]	2013	India	HIV-infected adults receiving ART at a treatment center.	Single-group pre-test post-test study	Participants received interactive voice response (IVR) calls twice a day for 1 month. Calls delivered messages on medication adherence, healthcare provider communication, nutrition, hygiene, active coping, positive cognitions, social support, relaxation, alcohol, condoms, STI, depression prevention, and alcohol use disorders. Additionally, three appointment reminder messages were sent at 7, 2, and 1 day prior to the scheduled follow-up assessment.	Any missed doses over past 3 days and prior weekend (Self-report: AACTG)[42]	Statistically significant change in proportions of participants with any missed doses from pre-test to post-test (39.1% baseline, 18.2% follow-up, test statistic not reported. p = 0.050).
			Participants were eligible if they were members of the Mamata Care and Treatment Center (MCTC) or the Mamata Network of Positive Women (MNPW), sex worker and community-based networks.			Missed dose score (ordinal measure of time elapsed since missing doses) (Self-report)	Statistically significant change in median missed dose score between baseline and follow up (median score 4 [1–2 weeks] baseline, median score 2 [1–3 months] follow-up, test statistic not reported, p = 0.015).
			N = 46				
Rodrigues et al., 2012 [70]	2010–2011	India	HIV-infected adults on ART 1 month at an infectious disease clinic.	Single-group pre-test post-test study	Participants received 1) An automated IVR call and 2) A non-interactive neutral picture SMS, once a week for 6 months. The IVR call asked participants about missed any doses in the past 24 hours and solicited responses. If a call was missed, three additional calls were made over the next 24 hours.	≥95% adherence (Pill count)	Proportion with adherence ≥95% at baseline, 1, 3, 6, and 12 months: 85%, 94%, 93%, 95%, 94%.
							Statistically significant change in proportion of participants with adherence ≥95% from baseline to 6 month follow-up (test statistics not reported, p = 0.016).
			Participants were eligible if they had mobile phone access.				
			N = 150				
Uzma et al., 2011 [71]	2008	Pakistan	HIV-infected adults on ART for at least 3 months prior to enrolment and receiving care at an HIV treatment center	RCT	Combined dosing schedule and phone reminder intervention delivered over 4 weeks. Participants were involved in developing personalized ART dosing schedules and received counseling to associate pill taking with routine daily activities. Participants also received weekly phone calls reminding them of their interaction with study staff and requesting strict adherence to their dosing schedules.	≥95% adherence (Self-report)	Statistically significantly higher proportion of participants with ≥95% adherence at 2 week follow-up in intervention compared to the control group (test statistic not reported, p = 0.000).
			N = 76 (38 intervention, 38 control)			Viral suppression (VL <50 copies)	Statistically significantly higher proportion of participants with viral suppression at 2 week follow-up in intervention compared to the control group (test statistic not reported, p = 0.012).
					Control: Routine adherence counseling and standard of care (not described).		
Shet et al., 2014 [72]	2010–2011	India	HIV-infected ART-naïve adults eligible to initiate ART at ambulatory clinics.	RCT	Customized motivational IVR calls sent on a weekly basis to participants for 96 weeks. Automated phone calls included a statement with a greeting and a hope that the participant was feeling well, and a question of whether the participant had taken the previous day's doses as prescribed. Non-interactive neutral pictorial messages were sent to participants 4 days after each automated call to serve as a reminder.	<95% adherence (Pill count)	Rates of <95% adherence over the study period were similar between intervention and control groups (aIRR = 1.24 [0.94, 1.63]).
						Virologic failure (VL >400 copies/mL)	Rates of virologic failure 6 months after ART initiation were similar between intervention and control groups (aHR = 0.96 [0.65, 1.43]).
			N = 631 (315 intervention, 316 control)				
					Control: Standard of care including up to three pre-ART counseling sessions, clinical assessments every 6 months, and ART distribution every 1–3 months.		
Maduka and Obin-West, 2013 [73]	2011	Nigeria	HIV-infected adults on ART ≥3 months identified as non-adherent and receiving care at a hospital ART clinic. Non-adherence defined as having a history of < 95% adherence at time of enrollment.	RCT	Individual adherence counseling provided by trained junior resident doctors in four 45–60 minute sessions over 4 months. Pre-scripted SMS reminders containing information on adherence and a reminder to take ART medications were sent to participants twice a week for 4 months. A contact number was provided with all reminder messages and participants were encouraged to call or send SMS to the contact number to acknowledge their receipt of the message or indicate a need for further counseling.	≥95% adherence over past 7 days (Self-report)	A statistically significantly higher proportion of participants in the intervention group achieved ≥95% adherence compared to the control at endline (χ^2^ = 5.211, p = 0.022).
						CD4 count	There were no statistically significant differences in mean CD4 counts between intervention and control groups at baseline (U = 1.13, p = 0.130); at endline, mean CD4 count was statistically significantly higher in the intervention group compared to the control at follow-up (U = 2.44, p = 0.007).
			Participants were eligible if they owned a mobile phone and were living with no other disease requiring daily medication.		Control: Standard of care including group health education, occasional reminders from doctors and pharmacists to take medication, and quarterly CD4 count assessments.		
			N = 104 (52 intervention, 52 control)				
Chung et al., 2011 [74]	2006–2008	Kenya	HIV-infected ART-naïve adults receiving care at an infectious disease center.	RCT	Participants randomly assigned to adherence counseling alone, alarm device alone, combined counseling plus alarm, or control.	<80% adherence (Pill count: Pharmacy refill)	Marginally significant differences in rates of <80% adherence between those that received counseling compared to those that did not 18 months after ART initiation (p = 0.053).
			N = 362 (92 counseling, 91 alarm, 83 counseling and alarm, 96 control)		Individual adherence counseling provided by a trained counsellor in 3 sessions each lasting 30–45 minutes. Duration of counseling intervention not described. Counseling sessions followed a written standardized protocol and explored personal barriers to adherence, HIV and ART information, and overcoming practical and personal issues faced by taking ART. The alarm device was a small pocket digital alarm to be carried for 6 months and programmed to beep and flash at times medications were to be taken.	Virologic failure (VL >5,000 copies/mL)	Statistically significantly lower rates of virologic failure between those that received counseling compared to those that did not 18 months after ART initiation (p = 0.008).
							No statistically significant differences in rates of <80% adherence between those that received alarm reminders and those that did not 18 months after ART initiation (p = 0.7).
							No statistically significant differences in risk of virologic failure between those that received alarm reminders and those that did not 18 months after ART initiation (p = 1.0).
							*Note*: *Authors reported no interaction between counseling and alarm device; results presented separately (All who received counseling vs*. *control*, *all who received alarm device vs*. *control)*.
					Control: Standard of care including pharmacist counseling at ART initiation.		
Simoni et al., 2011 [75]	2006–2008	China	HIV-infected adults initiating ART at a hospital.	RCT	Participants chose an alarm device, adherence counseling, or combined alarm plus counseling. Participants choosing the alarm device could use their own cell phone or a small, battery-powered device that would sound an alarm at pre-set dosing times. Alarm reminders were delivered for 13 weeks. Counseling followed an adaptation of the Life-Steps protocol and was delivered by a nurse in three 1-hour sessions over 9 weeks. Content focused on education about ART, increasing motivation for adherence, creating medication schedules, developing reminder strategies, and seeking social support. Participants were allowed to invite a treatment partner to attend sessions and participate in modified joint counseling.	100% adherence (Self-report)	Odds of 100% adherence in the past 30 days over the course of the study were statistically significantly higher in the intervention group compared to the control (OR = 2.23 [1.05, 4.72]).
				*Note*: *Participants randomized to receive the intervention self-selected to receive one of the 3 intervention arms; therefore*, *random allocation did not occur*.			
			N = 70 (36 Intervention, 34 control)			Continuous adherence over past 7 days (EAMD: MEMSCap)	No statistically significant difference in changes in 7-day EAMD adherence over the study period between intervention and control groups (β = -0.12 [-1.23, 0.99]).
						Continuous adherence over past 30 days (EAMD: MEMSCap)	No statistically significant difference in changes in 30-day EAMD adherence over the study period between intervention and control groups (β = -0.05 [-1.05, 0.95]).
						CD4 count	No statistically significant difference in average CD4 count change from baseline to 25 weeks between intervention and control groups (β = -0.10 [-2.30, 2.09]).
						VL	No statistically significant differences in average log VL from baseline to 25 weeks between intervention and control groups (β = -0.005 [-0.03, 0.02]).
					Control: Standard of care including monthly ART pick-ups and ART education session at baseline and before ART initiation.		
***Community- and home-based strategies***							
Williams et al., 2014 [76]	2010–2012	China	HIV-infected adult patients initiating or already on ARV with a detectable VL and reporting <90% adherence at clinics.	RCT	Home-based intervention based on Freirean pedagogical theory conducted by nurses and peer educators delivered on a bi-weekly then monthly basis for 6 months. Participants discussed their ART adherence with nurses and peer educators at home visits that were delivered every two weeks for the first 3 months of the intervention, then once a month for the remaining 3 months. Study staff were available by phone between home visits.	≥90% adherence (Self-report: VAS) [46]	The intervention group had a statistically significantly higher proportion of people who were adherent (84% vs. 53%) in a multivariate analysis (p = 0.009).
							*Note*: *Authors state that analysis is multivariate although multivariable appears to more accurately describe the analytic approach*. *Time point of adjusted analysis not stated*.
			N = 110 (55 intervention, 55 control)				
					Control: Standard of care including pre-treatment adherence counseling and monthly clinic visits.		
Kipp et al., 2012 [77]	2006–2009	Uganda	HIV-infected adults receiving care at either a rural health center providing community-based care (intervention) or at a regional hospital (comparison).	Quasi-experimental study	Volunteer community members provided CBAS through weekly visits to assigned patients. During visits volunteers performed a pill count, assessed the presence of clinical problems or adverse reactions, referred patients that required specialized treatment, and provided adherence counseling. Patients were encouraged to identify a treatment supporter to provide daily adherence support. Volunteers distributed ARTs to participants monthly. The intervention was evaluated after 24 months.	Virologic suppression (VL <400 copies/ml)	Odds of achieving virologic suppression were statistically significantly higher among the intervention group compared to the hospital-based comparison at endline (aOR = 2.47 [1.01, 6.04]).
			N = 385 (185 intervention, 200 comparison)				
					Comparison: Patients receiving clinic-based care at a regional hospital. Patients attended monthly hospital visits with a physician or clinical officer.		
Franke et al., 2013 [78]	2007–2008	Rwanda	HIV-infected adults (≥21 years, mean age 37) that were ART-naïve and initiating ART in clinics in two rural districts. Comparison clinic-based ART sites were selected from a different district.	Prospective observational cohort study	Individuals receiving community-based accompaniment were visited daily in their homes by community health workers (CHWs). CHWs provided social support, monitored for adverse events, identified potential barriers to adherence, and provided DOT of all medications. Food rations were provided for the first 10 months of ART. Transportation stipends were provided for clinic visits. CHWs accompanied participants for the first 4 monthly visits, then as needed afterwards. Social workers conducted screening and provided financial or instrumental support as identified.	Viral suppression (VL <200 copies/mL)	Intervention group more likely to be retained with a suppressed viral load at 1 year than comparison group (aRR: 1.15 [1.03, 1.27]; p = 0.01)
						CD4 count	No significant difference in changes in absolute CD4 count from baseline to 1 year between groups (adjusted difference in CD4 count change, 21.7 cells/μL [−16.9, 60.3]; p = 0.27).
			N = 610 (304 intervention, 306 comparison)				
					Comparison: Standard clinic-based care including CD4 evaluation at ART initiation and every 6 months thereafter and encouragement to disclose HIV status to friends and family and identify a “treatment buddy.”		
Muñoz et al., 2011 [79]	2005–2007	Peru	HIV-infected ART-naïve adults about to initiate ART living in poverty and referred to a tertiary hospital for HIV care.	Quasi-experimental study with matched comparison group	Community-based Accompaniment with Supervised Antiretroviral (CASA) adherence program employed a team of trained nurses, field supervisors, and lay health workers to conduct home visits and provide DOT of all ART doses. DOT provision lasted for 12 months, with tapered provision during the last 2 months. CASA teams monitored for side effects and threats to treatment adherence, provided emotional and material support, helped coordinate appointments, and assess mental health and socioeconomic needs. Intervention also included peer support groups and a selective microfinance program.	>95% adherence over past month (modified ACTG instrument)	Statistically significant difference in mean self-reported adherence after 2 years between intervention (79.3% adherence) and control (44.1% adherence) groups (X^2^ = 15.3, p < 0.01).
						Virologic suppression (VL < 400 copies/mL)	CASA support was associated with higher rates of virologic suppression (aOR = 2.46 [1.03, 6.09]) after 2 years.
			N = 120 (60 intervention, 60 control)				
					Comparison: Matched group of HIV-infected adults enrolled in care and living in a neighboring health district receiving standard clinical services (not described).		
Nyamathi et al., 2012 [80]	2009–2011	India	HIV-infected adult (18–45 years) women receiving ART for ≥ 3 months and had CD4 counts > 100 cells/mm^3^ living in rural high-prevalence villages.	RCT	Volunteer women (Ashas) provided adherence support through weekly home visits, monitoring barriers to ART adherence, and counseling to address barriers to accessing health care or adhering to treatment. Ashas asked participants about side effects, provided basic education and counseling, promoted a healthy lifestyle, and linked women to community resources. As needed, they also accompanied women to the district hospital or to a psychologist, and provided counseling on coping strategies. Participants also received a 6-session educational program and monthly nutritional support.	Continuous adherence over past month (Pill count)	Participants in the intervention group took a statistically significantly higher proportion of their ART doses over the past month compared to the control group (Coefficient not reported, p < 0.001).
			N = 68 (34 intervention, 34 control)				
					Control: Matched group of participants receiving standard of care (not described) in addition to 6-session education, nutritional support, and visits by “control” Ashas that evaluated adherence but did not assist women overcome adherence barriers.		
Coker et al., 2015 [81]	2006–2008	Nigeria	HIV-infected, treatment-naïve adults who are enrolled in care at a hospital.	RCT	Participants were randomized to receive a multi-component community-based adherence support (CBAS) intervention alone, CBAS plus home visits by peer educators, or standard of care.	Virological suppression (VL <400 copies/ mL)	No statistically significant difference in odds of virologic suppression between CBAS only arm and control group at 18 month follow-up (OR = 0.80 (0.38, 1.70]).
							No statistically significant differences in odds of virologic suppression between CBAS plus home visit arm and control group at 18 month follow-up (OR = 0.90 [0.43, 2.02]).
			N = 600 (200 CBAS, 200 CBAS plus peer educators, 200 control)		CBAS consisted of daily medication reminders, delivered by watch alarm or calls from a peer educator; and a home-based treatment partner to perform DOT in the home. Peer educators, some of whom were currently on ART themselves, were given extensive training on how to provide support through home visits. The intervention was delivered over 9 months. Frequency of home visits not described.		
					Control: Standard of care including standardized HIV and pretreatment counseling.		
Fatti et al., 2012 [82]	2004–2011	South Africa	HIV-infected ART-naïve individuals (≥16 years, median age 35.1 years for CBAS, 34.6 control) initiating ART from NGO-supported hospitals and primary care facilities that had electronic clinical data collection systems and active patient advocate (PA) programs.	Prospective observational cohort study	Patient advocates (PAs) provided CBAS for patients through home visits. After the initial psychosocial home-based screening visit, PAs conducted weekly home visits for one month, supervised taking of medication, advised on medication storage, performed adherence checks, provided one-on-one counseling and health education, screened for opportunistic infections, and provided referrals to clinics if indicated. Stable patients were visited on at least a 3-monthly basis. Analysis examined outcomes over 5-year period.	Viral suppression (VL <400 copies/ mL)	Statistically significantly higher odds of achieving viral suppression in intervention group compared to control among patients on ART at each 6-month interval between 6 and 60 months (aOR = 1.22 [1.14, 1.30] among patients 6 months on ART; aOR = 2.66 [1.61, 4.40] among patients 60 months on ART).
			N = 66,953 (19,668 intervention, 47,285 comparison)		Comparison: Patients at the same facilities who did not receive CBAS by PAs. Comparison patients also received 3 pretreatment group HIV and ART education sessions. Patients at certain sites had access to adherence counsellors.		
Igumbor et al., 2011 [83]	2007 -	South Africa	HIV-infected adults, children, and adolescents (59% of participants 25–39 years old) receiving care at health facilities.	Retrospective cohort study	Trained patient advocates (PAs) provided psycho-social assessment to identify barriers to adherence, conduct pre-treatment initiation education, and provide adherence support services through follow-up and home visits. PA services were made available to all patients with PA programming; however, not all patients chose to take part in these services.	>95% adherence (Patient treatment pickup rate: Pharmacy refill data)	A statistically significantly higher proportion of patients with PAs (89%) attained a treatment pickup rate of >95% compared to the proportion of patients without PAs who achieved the 95% pickup rate (67%; X^2^ = 6.131; p = 0.021). Time period for outcome not reported.
			N = 540		Comparison: Patients who chose not to receive PA program services. Standard of care not described.		
Achieng et al., 2012 [40]	NR	Kenya	HIV-infected ART-naïve adults who completed an ART preparation course and were receiving care from a hospital.	Prospective cohort study	Home visits were conducted by CHWs within one month of initiating ART; CHWs evaluated barriers to care, patient adherence, and overall health status. Patient referrals to the clinic and follow-up home visits performed as needed. Patients and their treatment supporters ("treatment buddies") attended monthly support group meetings led by CHWs; discussion centered around successes and challenges related to ART. All patients attended clinic visits on a monthly basis for the first 6 months on ART. Unannounced pill counts were conducted by providers. Pharmacy counseling was provided after provider visits; patients met with a pharmacist to discuss challenges with taking medication and to follow-up with adherence issues identified during provider visits.	Continuous adherence (Pharmacy refill data)	Adherence was statistically significantly higher among those who participated in ≥3 support group meetings (90% vs. 83%, p < 0.05), and who had pill counts performed by their clinician (90% vs. 76%, p = 0.001) after one year on ART.
			HIV-positive women who had received PMTCT were not excluded from this study.				
			N = 301				
Achieng et al., 2013 [41]	NR	Kenya	Population defined in Acheing et al., 2012.	Prospective cohort study: Re-analysis of pill count data from Acheing et al., 2012	Intervention description found in Achieng et al., 2012.	Continuous adherence (Pharmacy refill data)	Mean adherence over the past 6 months increased significantly as number of unannounced pill counts increased (r = 0.21, p<0.01).
					Pill counts were conducted by CHWs, pharmacists, and physicians. Re-analysis includes data only from physician pill counts. Number of pill counts conducted over past 6 months evaluated as predictor.	Treatment failure (detectable VL; ART discontinuation; death; or loss to follow-up)	Time to treatment failure increased significantly by the number of pill counts performed (mean time = 220 days [0 counts], 438 days [1–3 counts], and 497 days [4–6 counts], p<0.01).
							Increased number of pill counts significantly associated with decreased treatment failure over 600 days (aHR = 0.69, p = 0.042).
Kunutsor et al., 2011 [84]	2010	Uganda	HIV-infected adults currently on ART at a hospital HIV clinic.	RCT	Participants received assistance from health workers to identify a treatment supporter (TS) who was aware of the participant’s HIV status and had gained the trust of participants. TSs were provided with structured educational materials and were instructed to remind the patient to take their medication, attend follow-up appointments, remember all test results and clinic history, and accompany the patient to support group meetings. The intervention lasted 28 weeks. TS meetings were held every 2+ weeks to address patient non-adherence, burn-out, and other barriers to treatment.	Continuous adherence (Pill count)	There were no statistically significant differences in mean adherence at endline between intervention (99.1%) and control (96.3%) groups (test statistic not reported, p>0.05).
			N = 174 (87 intervention, 87 control)				
						≥95% adherence (Pill count)	Odds of having ≥ 95% adherence at endline were statistically significantly higher in the intervention group (OR = 4.51 [1.22, 16.62], p = 0.027)
					Control: Standard of care including monthly clinic visits and health education and adherence counseling.		
Gross et al., 2015 [85]	2009–2011	Botswana, Brazil, Haiti, Peru, South Africa, Uganda, Zambia, Zimbabwe	HIV-infected adults with confirmed virologic failure (> 1000 copies/mL) after ≥ 16 weeks of ART that were patients at ACTG site clinics.	RCT	Participants identified treatment adherence partners, who monitored drug taking, assisted in reminding participant to take their drugs, provided positive social support, and served as liaison to the clinical site if the participant was unwilling or unable to ask for help with adherence barriers. Adherence partners performed DOT at least one dose per day, 5 days a week. Intervention lasted 24 weeks.	% of doses taken per yearly quarter (EAMD: MEMSCap): <70%, 70–79.9%, 80–89.9%, 90–95%, >95%	No statistically significant differences in distribution of adherence categories between intervention and control groups at 24 week follow-up (OR = 1.21 [0.80, 1.83]).
							No statistically significant difference in cumulative probability of virologic failure at 24 week follow-up between intervention and control group (weighted difference = -6.6% [-16.5%, 3.2%]).
			N = 257 (129 intervention, 128 control)		Control: Standard of care (not described) and treatment partner who only received basic HIV and health education information.	Virologic failure (VL >400 copies/mL)	
Hickey et al., 2015 [86]	2011–2012	Kenya	HIV-infected adult patients who had already initiated ART at a health center.	Quasi-experimental two-group pre-test post-test design	ART patients invited to form "microclinics" of close family members, friends, or other supportive individuals. Each microclinic was assigned a CHW coordinator; microlclinics attended 10 bi-weekly discussions sessions lasting 2–3 hours each over 5 months. Session content included HIV prevention and treatment and group support promotion. Participants invited to participate in voluntary group HIV testing, which allowed for microclinic members to disclose their status to one another.	Drug concentration (Nevirapine concentration in hair sample)	Participation in the microclinics was not associated with greater changes in mean drug concentration over 18 month follow-up period (β = 6.7 [-2.7, 16.1]).
			N = 369 (216 intervention, 153 control)		Comparison: Communities in sub-locations not receiving intervention. Standard of care not described.		
***Pharmacist counseling***							
Silveira et al., 2014 [87]	2006–2009	Brazil	HIV-infected adults receiving HIV care at a medical school in an urban area.	RCT	Pharmaceutical care intervention using the Dáder method [88] to improve patient adherence. Patients received structured counseling by pharmacists on their prescription regimens at the time of initial drug dispensing and at monthly refill visits for a total of 12 months. At each scheduled meeting, pharmacist and patient addressed, reviewed, and solved drug-related problems.	≥95% adherence over past 3 days (Self-report)	No statistically significant differences in proportions of participants with self-reported adherence ≥95% over past 3 days between intervention and control groups at endline (aRR = 1.05 [0.95, 1.15]).
			N = 332 (166 intervention, 166 control)			Undetectable VL (VL <50 copies/mL)	No statistically significant differences in rates of undetectable VL at endline between intervention and control groups (aRR = 1.08 [0.97, 1.20]).
					Control: Standard of care including monthly ART pickup and clinical visits every 4 months. Patients received information on medications from nurses and had no encounters with a pharmacist.		
***Depression treatment***							
Moosa, M. Y. H., and Jeenah, F. Y., 2012 [89]	2008	South Africa	HIV-infected adults who were stable on ART for ≥6 months receiving care at a university HIV research unit.	RCT	All patients received a clinical diagnostic evaluation and the Hamilton Depression rating scale (HAMD) at study entry; depressed patients were randomized to receive treatment (10–20 mg citalopram) or interpersonal psychotherapy (IPT). Duration of intervention not described.	Continuous adherence over past 3 days (Self report: Pills missed)	No statistically significant difference in change in mean adherence from baseline to endline between intervention and control groups (p>0.05).
			N = 62 (32 intervention, 30 control)		Control: Non-depressed patients receiving standard of care (not described).	Continuous adherence (Clinic-based pill count)	
***Facility-based interventions***							
Kunutsor et al., 2012 [90]	2009–2010	Uganda	HIV-infected adults on ART for ≥3 months at government facility ART sites.	Single-group pre-test post-test study	The intervention delivered an enhanced adherence package, which built upon the already existing health education, counseling, and systematic monitoring that was included in standard of care treatment. Components included strengthened individual counseling, expanded health education classes and leaflets, adherence diaries, mobile reminders, treatment supporters, tracing, adherence support workers training, and strengthened adherence monitoring. Duration of intervention not reported. Standard of care included monthly ART refills.	Continuous adherence (Combination of clinic-based pill count and self-report)	Mean adherence increased significantly over the study period (97.4% baseline, 99.1% endline, p = 0.001).
			N = 967			≥95% adherence (Clinic-based pill count and self-report) [91]	A statistically significantly greater proportion of participants achieved ≥95% adherence comparing endline to baseline (difference = 7.0% [4.6, 9.4] p = 0.001).
Obua et al., 2014 [92]	2009–2010	Uganda	HIV-infected adults receiving care from district-level hospitals with poor patient flow or congestion-related characteristics.	Single-group pre-test post-test study	Intervention introduced an appointment system, which consisted of a written record of follow-up appointments, encouraging patients to return based on that record, and appointment diaries to remind patients of their next visit. If patients reported no problems at triage, providers could “fast track” patients who had been on ART for at least 12 months, had a self-reported adherence ≥95%, and only needed a drug refill. Prescribers at health facilities were also encouraged to give longer prescriptions (increasing from 30 days to 60 or 90) among patients that were ≥95% adherent to reduce the number of refills needed. The intervention was implemented over a 17 month period.	Experienced cohort: ≥3 days without medication (Pharmacy refill data)	Experienced cohort participants had statistically significantly lower odds of having ≥3 days without medication comparing from post-intervention to pre-intervention (aOR = 0.69 [0.60, 0.79]).
							Newly-treated cohort participants had a statistically significantly lower probability of having ≥7 days without medication comparing from post-intervention to pre-intervention (p<0.0001).
			N = 1,481			Newly-treated cohort: ≥7 days without medication (Pharmacy refill data)	Newly-treated cohort participants had a statistically significantly lower probability of having ≥14 days without medication from post-intervention to pre-intervention (p<0.0022).
			Two cohorts were selected: Treatment experienced cohort (on ART ≥1 year, adherence ≥95%, n = 720), and newly-treated cohort (initiating ART during study period, n = 761).				
						Newly treated cohort: ≥14 days without medication (Pharmacy refill data)	
Boruett et al., 2013 [93]	2008–2009	Kenya	HIV-infected adults receiving care from rural district hospitals.	Quasi-experimental two-group pre-test post-test study	Intervention consisted of: 1) A clinic appointment diary to record scheduled and actual appointment dates for each patient and monitor facility appointment-keeping performance; 2) Patient monitoring forms were changed so clinicians would ask about missed ART doses at each visit. Patients that reported missed doses were identified as non-adherent and received additional adherence counseling; 3) Staff received targeted training on adherence, adherence interventions, and data collection and analysis; 4) Study staff visited facility teams to support implementation. Intervention was evaluated after 13 months.	100% adherence over past 3 days (Self-report)	>90% of participants in intervention and control groups reported 100% 3-day adherence at pre- and post-intervention.
			N = 1894			Medication gap of more than 14 days (Pharmacy refill and clinic attendance records).	No significant difference in 14-day medication supply gaps over the study period between intervention and comparison for either experienced or newly treated cohorts.
			Two cohorts were selected: Experienced cohort (on ART ≥ 1 year, intervention = 446; comparison = 352), and newly-treated cohort (on ART < 1 year, intervention 520, comparison = 576).				
					Comparison: Standard of care including clinic visits varying from every month to every 3 months for clinically stable patients.		
***Nutrition support***							
Tirivayi et al., 2012 [94]	2009	Zambia	HIV-infected adults receiving care from beneficiary clinics.	Quasi-experimental study	Participants received nutritional support for 12 months, which included monthly rations of maize, vegetable oil, peas, and a corn/soy flour blend. The monthly ration had a marked value of approximately US $18 in 2009.	Continuous adherence (Medication possession ratio: Pharmacy refill data)	Mean adherence was statistically significantly higher at 6 months in the intervention group compared to a propensity score matched comparison (t = 4.06, p<0.01).
							*Note*: *No baseline measures of adherence were available*.
			Patients were eligible if they scored above the food insecurity cut-off.		Comparison: Patients receiving care from clinics that did not distribute food rations. Standard of care not described.		
			N = 145				
Serrano et al., 2010 [95]	2007	Niger	HIV-infected patients receiving ART at an ambulatory treatment center. Median age 38 years intervention group, 38.5 comparison.	Retrospective cohort study	Eligible participants received a monthly family food ration for 6 months and nutritional advice to increase food intake during ART treatment during a follow- consultation. Rations were calculated based on family size and consisted of cereal, legumes, and Vitamin A-fortified vegetable oil.	Continuous adherence (Pill count and self-report)	Intervention was significantly associated with higher mean adherence at endline (test statistic not reported, p<0.005).
						CD4 count	Intervention was significantly associated with higher increases in CD4 counts from baseline to endline (aRR = 43.0 [4.5, 81.5]).
			Participants were eligible if they had CD4 count <200 cells/mm^3^, advanced clinical stage of AIDS, or BMI <18.5 kg/m^2^.		Comparison: Retrospectively selected patients receiving care from the same clinic the year before. Patients received nutritional advice but no food supplementation and attended monthly clinic visits.		
			N = 180 (62 intervention, 118 comparison)				
Martinez et al., 2014 [96]	2009–2011	Honduras	HIV-infected adults on ART for ≥ 6 months with a history of sub-optimal adherence receiving care from large hospitals and small hospitals. Suboptimal adherence indicated by missed clinic appointments, delayed pharmacy refills, or self-reported missing medication doses.	Cluster RCT	Participants received a monthly household food basket for 1 year which included food for a household of 5 and contained maize, rice, beans, fortified corn-soy blend, and vegetable oil. The basket’s marked value was approximately US$46. Participants received nutrition education, based on the information-motivation-behavioral skills model, and consisted of monthly 20-minute individual counseling sessions and (5) 1-hour group sessions with cooking activities and practical demonstrations delivered over 6 months.	>1 week late in refilling any monthly ART prescription in past 6 months (Pharmacy refill data)	Intervention had no significant effect on missed appointments or self-reported missed ART doses at 6 or 12 months.
							Intervention was significantly associated with a reduction in delayed pharmacy refills at 6 months (β = -0.196, p < 0.01) compared to control, but not at 12 months.
						Any missed ART doses in past month (Self-report)	
					Control: Participants at control sites received the nutrition education component and were given the food basket when data collection completed. Standard of care not described.		
			Participants were eligible if they were underweight and/or demonstrated household food insecurity.				
			N = 400 (203 intervention, 197 control)				
Ivers et al., 2014 [97]	2010–2011	Haiti	HIV-positive adults initiating ART within 24 months, living in the program catchment area, and receiving care from health centers.	RCT	Participants received monthly rations of a peanut-based ready-to-use supplementary food (RUSF), distributed during monthly HIV clinic attendance or delivered by CHW as needed. Rations consisted of an “individual ration,” targeting the HIV patient, and a “family ration” to offset the monthly needs of participants’ families. Intervention was evaluated after 12 months.	Missing any doses in past 30 days (Self-report)	No statistically significant differences in changes in missed doses from baseline to 6 or 12 months between intervention and control groups (test statistic not reported, p = 0.61 at 6 months, p = 0.52 at 12 months).
			N = 524 (285 intervention, 239 control)		Control: Received individual and family rations of a corn-soy blend plus (CSB+) with fortified oil and sugar with similar caloric composition as the peanut-based RUSF. Standard of care not described.		
***Financial support***							
Peltzer, 2012 [98]	2007–2010	South Africa	HIV-infected, ART-naïve adults about to initiate ART at hospitals.	Prospective cohort study	Evaluated the impact of South Africa's disability grants (DG), which help financially support some HIV-infected individuals until they are healthy enough to re-enter the workforce. Typical eligibility for receiving a DG was CD4 count ≤200 cells/mm^3^.	≥95% adherence (Self-report: VAS) [46]	No statistically significant difference in odds of adherence ≥95% after 20 months on ART between intervention and comparison groups (OR = 4.57 [0.61, 34.10]).
			N = 735		Comparison: Participants who did not receive a DG. Standard of care not described.		
***Task shifting***							
Fairall et al., 2012 [99]	2008–2010	South Africa	HIV-infected adults on ART ≥6 months and on treatment at enrolment, receiving care at nurse-led ART clinics or at doctor-led referral hospitals.	RCT	Intervention clinics and hospitals implemented the Streamlining Tasks and Roles to Expand Treatment and Care for HIV (STRETCH) program that trained to nurses to initiate and re-prescribe ART for patients that met certain criteria (For re-prescription patients included in the analysis: Undetectable viral load; no severe side-effects; no new opportunistic infections). STRETCH was implemented alongside PALSA PLUS guidelines for monitoring and referral of ART patients. Each intervention facility also established management support teams.	Viral suppression (VL <400 copies/mL)	The intervention was not associated with a statistically significantly higher risk of achieving viral suppression 12 months after enrollment compared to the control (Risk difference = 1.1% [-2.3%, 4.6%]).
						CD4 count	Intervention participants had statistically significantly higher mean CD4 counts at follow-up compared to control participants (β = 24.2 [7.2, 41.3] p = 0.007). Mean follow-up time 18 months.
			N = 5,238 patients in 31 clinics (16 intervention clinics with 2583 patients, 15 control clinics with 2656 patients)				
					Control: Standard of care. Nurse-led clinics established ART eligibility and referred patients to doctor-led hospitals for treatment initiation or re-prescription. Patients returned to hospitals every 3–6 months for prescription review by a doctor. Drugs delivered to clinic sites.		
Kiweewa et al., 2013 [100]	2007–2009	Uganda	HIV-infected adult women that were ART-naïve, pre-partum or postpartum and referred by an affiliated PMTCT program to receive care at a Hospital ART clinic.	RCT	Patients initiated ART and attended 2- and 12-month follow-up appointments with a doctor and certified counsellor. Patients visited with only a nurse and peer counsellor during remaining follow-up appointments at week 2 and months 1,3,6, and 9. Peer counsellors were clinic patients currently on ART trained in basic counseling who also conducted home visits if participants missed an appointment. Outcomes were evaluated after 6–12 months.	Virologic success (VL <400 copies/mL)	Rates of virologic success were comparable between the intervention and control groups (-3% difference [-12%, 11%, within 10% non-inferiority cut-off]).
						Continuous adherence (Pill count)	Cumulative probabilities of virologic success were similar between intervention and control groups (p = 0.733).
							Mean adherence was similar in intervention and control groups (99% adherence 97% control, difference 2% [-1%, 5%, within 10% non-inferiority cut-off]).
			N = 92 (48 intervention, 44 control)		Control: Standard of care. Participants received all care from a doctor and a certified counsellor; and had appointments at weeks 2 and 4, and on a monthly basis afterward.		
Selke et al., 2010 [101]	2006–2008	Kenya	HIV-infected adults receiving care from a health center.	RCT	Trained Community Care Coordinators (CCCs) conducted home visits with patients. CCCs entered patient symptom data, vital signs, and ART adherence assessments (derived from in-home pill counts) into PDAs. Pre-programmed alerts, triggered if specified parameters were met, prompted CCCs to either return the next day to re-evaluate the patient, transport the patient to the clinic for urgent evaluation, or call the clinical officer for consultation. If no immediate alerts were triggered, CCCs dispensed a one-month supply of the patient’s medications from a prefilled kit. Patients also had scheduled clinic visits every 3 months.	Proportion of participants with >95% adherence (Pill count)	Comparable proportions of participants with >95% adherence at 6 months (96% intervention, 97% control, p = 0.47) or at 12 months (94% intervention, 97% control, p = 0.47) between intervention and control groups.
			Patients were eligible for enrolment if they were stable on ART for ≥ 3 months with no adherence issues.			Detectable VL (VL >50 copies/mL)	Comparable % of participants with VL detection between intervention and control groups at 12 months (10.5% intervention 13.5% control, p = 0.65).
						CD4 count	Comparable mean CD4 cell counts at 6 months (354 cells/mL intervention, 306 control, p = 0.24 and 6 months) and 12 months (404 intervention, 358 control, p = 0.50 at 12 months) between intervention and control groups.
			N = 208 (96 intervention, 112 control)				
					Control: Standard of care, which involved nurse vital recording and triage. Physicians and medical officers covered the remaining HIV-related care.		
***Decentralization***							
Gorman et al., 2015 [102]	2002–2007	Kenya	HIV-infected individuals receiving care from semi-mobile sites or a district hospital.	Retrospective cohort study	After registering at the district hospital, newly diagnosed individuals with HIV could choose to continue care at the hospital or at a semi-mobile clinic, which provided clinical monitoring, support groups, food distribution, and testing and treatment for opportunistic infections. A clinical team of at least a nurse, a clinical officer, and a social worker from the district hospital travelled once weekly to one of the four semi-mobile clinic sites.	CD4 count	No statistically significant difference in mean CD4 count between intervention and comparison groups after a mean of 12 years (t = -0.11, p = 0.91).
						100% adherence (Pill count)	No statistically significant difference in proportions of patients with 100% adherence between intervention and comparison groups after a mean of 12 years (χ^2^ = 4.54, p = 0.34).
			Age of eligibility not reported; mean age 33.5 years hospital-based cohort, 36.0 years semi-mobile clinic cohort.				
			N = NR		Comparison: Patients continue to receive care from a district hospital.		

### Counseling interventions

12 studies described counseling interventions that were delivered to individuals, groups, or combined individual and group sessions [47–52, 54, 56–58, 60, 61]. Two studies were conducted among adolescents only [58, 60], two among both adults and adolescents [50, 61], and eight among adults only [47–49, 51, 52, 54, 56, 57]. Three additional studies that combined individual counseling with SMS reminders are described in the following section.

### Individual adherence counseling

We identified four studies (two RCTs, one single-group pre-test/post-test study, and one retrospective cohort study) that evaluated individual adherence counseling interventions; three of these were conducted among adults only [47–49] and one among both adults and adolescents [50]. Counseling sessions were held by lay health workers, trained health professionals, or multidisciplinary teams, and aimed to increase HIV knowledge and address adherence barriers. None of the studies found statistically significant effects on adherence. All studies were fair to poor quality, and faced methodological issues such as high refusal rates (>20%) [47], inconsistent intervention implementation [49], and large amounts of missing data [50]. Two publications failed to report sample size calculations or power analyses [49, 50].

### Group adherence counseling

Group counseling interventions were described in seven studies, five of which were conducted among adults [51, 52, 54, 56, 57] and two among adolescents [58, 60].

Among adults, statistically significant results were found in only one of the five studies, which were overall of fair quality. A quasi-experimental pilot study in Nigeria found significant differences in mean adherence (Z = -3.581, p<0.001) and three other adherence outcomes between adult women receiving a group motivational interviewing intervention and women that did not [51]; however, this evidence is limited because baseline measures were not presented. An RCT in Zambia found significant improvements in adherence among adults receiving a group counseling intervention compared to individual counseling; however, these differences were no longer detectable once the groups were crossed over, and adherence decreased in both groups [52]. Based on these studies, evidence supporting group counseling as a strategy to improve adherence among adults is currently lacking.

**Fig 2:**
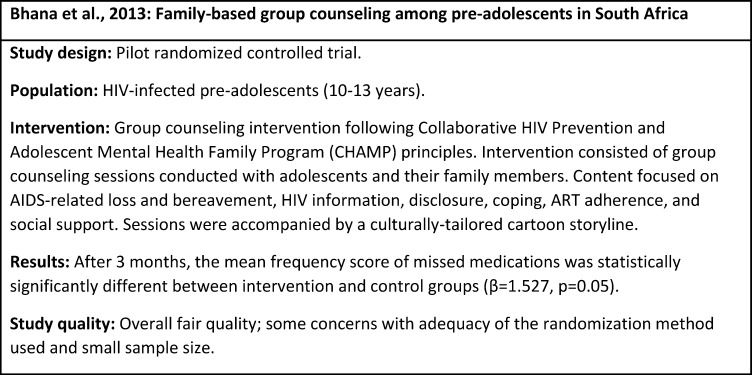
Bhana et al., 2013.

**Fig 3:**
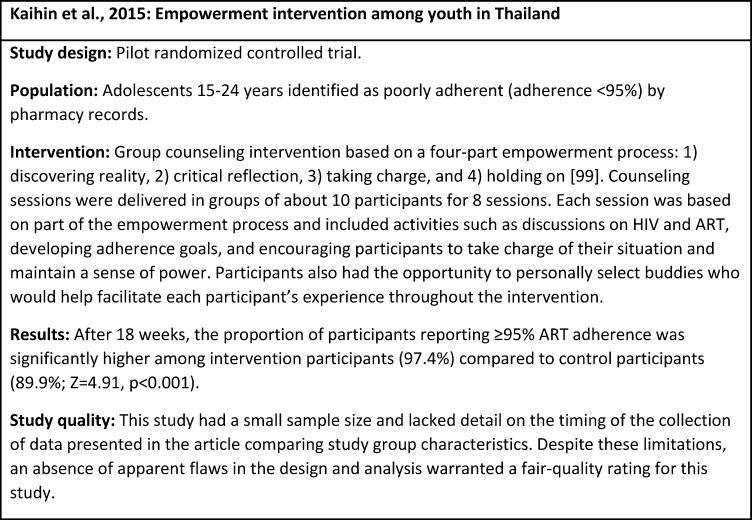
Kaihin et al., 2015.

Among the two studies that evaluated group counseling for ALHIV (Figs 2 and 3), one found statistically significant intervention effects [58] and the other did not [60]. A pilot RCT in Thailand delivered group counseling to adolescents (15–24 years) and found significant differences in the proportions of patients >95% adherent to ART at endline (χ^2^ = 14.723, p<0.001) [58]. A second pilot RCT in South Africa evaluated a group counseling intervention delivered to young ALHIV (10–13 years) and their family members and did not find a significant treatment effect (β = 1.527, p = 0.05) [60]. It is difficult to draw conclusions based on two studies with small sample sizes; however, the presence of some significant findings for the effect of group counseling on adolescent adherence shows that further investigation is warranted.

### Individual plus group adherence counseling

An RCT in Indonesia used a psychiatrist to deliver both individual and group counseling sessions to adult women and found no statistically significant effects on self-reported adherence or viral load [61]. The validity of these findings are uncertain given the authors’ failure to describe the control group or report p-values for outcome analyses, and reporting of point estimates outside of reported confidence intervals.

### mHealth interventions

Thirteen studies described interventions that included the use of mobile phones as a platform to improve adherence [63–75]; 12 of these studies were conducted among adults [63–67, 69–75] and one was conducted among adults and adolescents combined [68]. Interventions included SMS reminder messages sent at regular intervals or triggered by EAMDs, interactive voice response (IVR) phone calls, and multi-faceted interventions using SMS reminders combined with adherence counseling.

### SMS reminders

Four RCTs conducted among adults tested interventions that reminded participants to take their medications by sending SMS messages at regular intervals [63–66]. A multi-site RCT in Kenya found that receiving weekly SMS that solicited responses from participants decreased rates of non-adherence (RR = 0.81 [0.69, 0.94]) and virologic failure (RR = 0.85 [0.72, 0.99]) among intervention participants compared to a standard-of-care control [63]. Another RCT in Kenya compared each of four study arms–short daily messages, short weekly messages, long daily messages, and long weekly messages–to a control of no SMS reminders and found no statistically significant effects [64]. Additional analyses revealed that weekly delivery of SMS (whether short or long) was associated with improved adherence compared to the control (p = 0.03), but no effect was observed for other combined groups of daily, short, or long reminders [64]. The remaining two studies found no effect on adherence [65, 66]. The mixed results of these four studies—which were overall fair quality—provides no clear support for the effectiveness of SMS reminders to improve adherence. Statistically significant results from the study that asked participants to respond to the SMS rather than sending “one-way” messages indicate that further investigation into the effectiveness of this strategy is warranted.

### EAMD-triggered SMS reminders

Two good-quality RCTs evaluated interventions that delivered triggered SMS reminders when EAMDs were not opened during scheduled dosing periods [67, 68]. An RCT conducted among adults initiating ART in China found statistically significant differences between intervention and control groups in the likelihood of achieving ≥95% adherence (RR = 1.69 [1.29, 2.21]) and mean adherence (p = 0.003) post-intervention [67]. The study did not find significant differences in virologic outcomes, which may be attributable to the relatively short follow-up time (6 months) or to high rates of viral suppression in the control group (98%) at baseline. Another RCT conducted with adults and adolescents in South Africa found no significant effects on adherence or virologic failure after 6 months on the intervention [68]. Given the mixed findings of these two studies, more research is needed to better explore the potential of EAMD-triggered SMS reminders.

### IVR or phone call reminders

Four studies conducted among adults tested the use of IVR calls or phone calls as reminders to improve adherence [69–72]; three of these found statistically significant results [69–71]. A single-group pre-test/post-test study in India that provided twice-daily IVR calls as well as SMS appointment reminders found significant increases in time elapsed since participants missed a dose (p = 0.015) from baseline to post-intervention [69]. Another single-group pre-test/post-test study in India that examined the effect of weekly IVR calls combined with picture SMS found a significant increase from baseline to post-intervention in the proportion of participants with ≥95% adherence (85% to 94%, p = 0.016) [70]; however, the study had a substantial (36%) refusal rate. An RCT in Pakistan combined a patient-designed ART dosing schedule with weekly reminder phone calls and found significant differences in proportions of patients reaching optimal adherence (reported as p = 0.000) and viral suppression (p = 0.012) between intervention and control groups [71]. The fourth study examined the effect of IVR calls on adherence and did not find significant intervention effects [72]. Despite significant results from three out of the four studies, the strength of the evidence is limited by nonexperimental study designs [69, 70] and methodological issues such as high refusal rates [70] and short intervention and follow-up times [69].

### SMS or alarm reminders plus individual adherence counseling

Three studies found mixed results on the effect of SMS or alarm device reminders combined with individual adherence counseling for adults [73–75]. An RCT in Nigeria examined the effects of individual adherence counseling and twice-weekly SMS reminders for non-adherent adults and found significant differences in the number of participants who achieved ≥95% self-reported adherence (χ^2^ = 5.211, p = 0.022) and in mean CD4 cell count (Mann-Whitney U-test, U = 2.44, p = 0.007) between intervention and control groups at endline [73]. However, the proportion of participants achieving adequate adherence was still sub-optimal (76.9% intervention, 55.8% control). A four-arm RCT in Kenya compared adherence improvements and rates of virologic failure between participants receiving individual adherence counseling, alarm reminders, or both counseling and reminders and patients receiving standard of care; the study only found statistically significant differences in virologic failure rates (p = 0.008) between participants who received adherence counseling and those who did not regardless of receiving reminders [74]. A third RCT in China allowed participants to self-select into one of three intervention conditions (alarm device, adherence counseling, or alarm device plus counseling) and compared all intervention participants to a control group receiving adherence education; the study found positive results on self-reported adherence (OR = 2.23 [1.05, 4.72]) and did not find any effect on clinical adherence measures [75]. Given the mixed results and methodological quality issues of these studies, better-designed and -implemented studies should be conducted before this strategy's effectiveness can be determined.

### Community- and home-based interventions

Thirteen studies described 12 interventions implemented in participants’ communities or homes [40, 41, 76–86]. These interventions included adherence support provided by lay health workers or volunteers, community- and facility-based adherence activities, home-based directly observed therapy (DOT) or adherence support by lay treatment supporters, and community-based social support. Two studies were conducted among both adults and adolescents [82, 83]; the remaining 11 were conducted among adults [40, 41, 76–81, 84–86].

### Community-based adherence support

Eight studies (three RCTs, two quasi-experimental studies, and one prospective and two retrospective cohort studies) tested or examined associations between adherence outcomes and community-based adherence support (CBAS) interventions [76–83]. Six studies were conducted among adults [76–81] and two included adults and adolescents [82, 83]. CBAS interventions included adherence support through home visits by a community-based health worker or volunteer and included activities such as DOT, basic clinical assessments, referrals, pill counts, and home ART delivery.

Among the six overall fair-quality CBAS intervention studies conducted among adults [76–81], five had statistically significant results [76–80]. An RCT in China provided home visits by nurses and peer educators to non-adherent adults and found that greater proportions of patients achieved ≥90% adherence over the intervention period compared to the control (84% intervention, 53% control, p = 0.009) in adjusted analyses [76]; however, this study was limited by high loss to follow-up in the control group. A quasi-experimental study in Uganda provided weekly home visits by volunteer community members to perform pill counts, deliver ARTs, and assess clinical problems and provide referrals; compared clinic-based patients, participants had significantly higher odds of achieving virologic suppression (OR = 2.47 [1.01, 6.04]) [77]. A prospective observational cohort study compared patients receiving weekly home visits by patient advocates to patients receiving clinic-based care and found that patients receiving home visits were significantly more likely to be retained in care with a suppressed viral load after one year (aRR = 1.15 [1.03, 1.27]) [78]. Two additional studies found positive results [79, 80]; but were limited by selection bias [80] and differential attrition [79]. Despite some methodological limitations, current evidence suggests this intervention strategy warrants exploration.

Two retrospective cohort studies conducted among adults and adolescents found statistically significant associations between exposure to CBAS and adherence [82, 83]. A study in South Africa examined exposure to Patient Advocates (PA) as part of a CBAS project and observed higher odds of achieving viral suppression among patients who had PAs assigned to them compared to those who did not (aOR = 1.22 [1.14, 1.30] at 6 months; aOR = 2.66 [1.61, 4.4] at 5 years) [82]; however, these findings were limited by incomplete data and no measurement of the exposure or frequency of interactions with PAs. A second study in South Africa also compared adherence rates by exposure to PAs among patients and observed significantly higher rates of adequate adherence (X^2^ = 6.131; p = 0.021) among those with PAs compared to those who did not [83]; however, the evidence is weakened by selection bias as study sites were selected based on the completeness of data available. These two observational studies provide initial evidence that assignment to PAs may improve adherence, but methodological problems limit confidence in their results.

### Multi-component facility- and community-based program

Two publications described one observational study that evaluated associations between program uptake and treatment outcomes among adults in Kenya [40, 41]. Program components included home visits by CHWs, treatment supporters, support groups, clinician pill counts, and pharmacist counseling. One publication reported significantly higher adherence among those who had participated in more than three support group meetings (p<0.05) and those who had four or more unannounced clinician pill counts (p = 0.001) compared to those who did not [40]. The second publication found a positive linear relationship between adherence and the number of unannounced pill counts performed (r = 0.21, p<0.01) [41].

### Peer treatment supporters

Two RCTs evaluated peer treatment supporters that provided adherence reminders or DOT to adult patients [84, 85]. An RCT in Uganda found a significantly higher proportion of participants receiving adherence reminders from treatment supporters were ≥95% adherent compared to the control at endline (OR = 4.51 [1.22, 16.62], p = 0.027), but found no significant differences in mean adherence [84]. The second RCT, conducted among adults across sites in eight countries, tested the effect of DOT provided by peer treatment supporters but found no significant effects on adherence or virologic failure among adults on second-line ART [85]. Although both studies had randomized designs and were of good quality, the lack of consistent results and the small number of studies indicate the need for more research to determine the effectiveness of this strategy.

### Community-based social network support

A quasi-experimental study in Kenya examined impacts on ART adherence of a community-based social network support intervention that provided education and social support to groups consisting of one HIV-infected individual and his or her close friends or family members [86]; no significant differences in hair ART concentrations were observed between the intervention and comparison groups. However, it should be noted that there were markedly higher refusal rates among the intervention group than in the comparison group, and that results may have been influenced by contamination between study groups.

### Pharmacist counseling

One RCT tested a structured counseling intervention delivered by pharmacists to adult ART patients in Brazil that had no effects on self-reported adherence or viral load [87]. The authors attributed the lack of significant findings to high baseline adherence among study participants.

### Depression treatment

An RCT in South Africa randomized HIV-infected adult patients with clinically diagnosed depression to receive either pharmacological treatment for depression or interpersonal psychotherapy and compared changes in patients’ adherence to patients without depression receiving standard of care [89]. There were no significant differences between either treatment arm and the control group, or between the two treatment arms. This study is limited by differences in baseline characteristics of individuals in the intervention and control groups as well as by a small sample size (n = 30 control group, 32 intervention group).

### Facility-based interventions

Three studies examined the impact of facility-based interventions to increase adult patients’ ART adherence through strengthened patient services, support services such as adherence reminders, and changes to staff training and clinic workflow [90, 92, 93]; two of these studies reported statistically significant effects [90, 92]. A single-group, pre-test/post-test study in Uganda tested an enhanced adherence package that provided improved counseling, health education, adherence diaries, mobile reminders, treatment supporters, tracing, and strengthened adherence monitoring [90]. The study found that participants experienced a significant increase in mean adherence (97.4% to 99.1%, p<0.001) and that the proportion of participants with ≥95% adherence increased over time (7.0% [4.6, 9.4] p = 0.001). Another single-group, pre-test/post-test study in Uganda introduced a new appointment system, provided appointment reminders, encouraged providers to give longer prescriptions to reduce refill frequency, and “fast tracked” stable patients needing ART refills [92]. The study found a significant decrease in the odds having a gap in taking medication over the past 3 days comparing pre- to post-intervention (aOR = 0.69 [0.60, 0.79]). A third, quasi-experimental study that examined increased adherence monitoring and targeted adherence counseling for non-adherent patients did not find significant intervention effects; however, baseline adherence was high (>90%) in both study groups [93]. Although two studies found statistically significant results, their non-experimental study designs limit the strength of these findings.

### Instrumental support interventions

Five interventions tested the effect of instrumental (tangible) support on ART adherence among adults [94–98]. Four of these examined nutrition support through monthly food rations [94–97], and one evaluated the provision of disability grants to people living with HIV [98].

### Nutrition support

Four studies (two RCTs, one quasi-experimental study, and one retrospective cohort study) evaluated nutrition-support interventions [94–97]; three found statistically significant effects on adherence [94–96]. A quasi-experimental study in Zambia found significantly different estimates of mean adherence at endline between intervention and comparison groups (t = 4.06, p<0.01) [94]. Significant positive results were also found in a retrospective cohort study in Niger that examined associations between exposure to a monthly food ration and mean adherence (p<0.005) and mean CD4 counts (aRR = 43.0 [4.5, 81.5]) [95]. These findings are limited by their observational nature and the fact that no baseline adherence or clinical measures were reported for the exposure groups. The provision of a monthly household food basket was also examined in an RCT in Honduras [96] that found that participants receiving nutritional education plus the food basket had fewer delayed pharmacy refills than those receiving education only (β = -0.196, p<0.01) but did not have any effect on self-reported adherence or missed appointments. The fourth study, an RCT in Haiti, compared a standard ready-to-use supplementary food to a less expensive corn-soy blend on a variety of clinical outcomes including adherence [97]; over the 12-month intervention, adherence did not change significantly in either group nor were there significant differences between the two groups’ adherence at any time point (0, 6, and 12 months). Positive effects from the three studies that examined adding nutrition support to ART care provide preliminary evidence for the use of this strategy to improve adherence among adult patients.

### Disability grants

One prospective cohort study examined the relationship between adherence and receiving disability grants among adult patients initiating ART in South Africa [98]. The authors compared self-reported adherence between patients continuing to receive a disability grant and those that had received a grant and later lost their eligibility status and found no statistically significant association, likely due to the fact that over 90% of study participants maintained >95% adherence while receiving and after losing the grants.

### Task-shifting and decentralization interventions

Four studies tested ART service delivery interventions for adult patients [99–102]; three of these evaluated task-shifting of services from physicians to lay health workers, nurses, or peer counsellors [99–101], and one evaluated providing decentralized services at semi-mobile clinics [102].

### Task shifting

Three RCTs evaluated task-shifting of ART care and found equivalent or improved adherence outcomes for adult patients who received ART services from lay health workers, nurses, or peer counsellors compared to those who remained in standard care delivered by a physician or clinical officer [99–101]. A study in South Africa found comparable rates of viral suppression and significantly higher mean CD4 counts at follow-up for participants who visited trained nurses for ART re-prescription compared to doctors (β = 24.2 [7.2, 41.3], p = 0.007) [99]. The other two studies, one which shifted services from doctors and certified counsellors to nurses and peer counsellors, and the other which provided care by trained lay health workers, demonstrated that services provided by lower cadre or lay health providers were not inferior to standard of care services [100, 101]. Two of the interventions required patients to be stable on ART for a defined period before they were eligible for task-shifted services [99, 101], which may limit the utility of this intervention for individuals initiating ART or experiencing clinical or adherence issues. Although limited in number, the three task-shifting studies provide promising indications that stable patients who are down-referred for ART care experience equivalent, if not improved, adherence compared to standard care.

### Decentralization

One retrospective cohort study in Kenya examined the association between receiving care at a decentralized, semi-mobile clinic or a district hospital and adherence and CD4 cell counts [102]. This study found no statistically significant associations between where patients received care and their mean CD4 count or pill-count adherence; high proportions of patients reported taking all of their medication (81% of hospital-based patients, 86% of semi-mobile clinic patients).

### Limitations of the reviewed studies

The quality of the studies included in this review varied; fewer than one-third of all studies achieved a “good” quality rating, and nearly all of these were RCTs. Our assessment of a study’s quality was often limited due to a failure to report critical information such as sample size or power calculations or participant inclusion criteria, or to adequately describe analyses or measures. Moreover, a substantial number of studies were affected by large, and often differential, attrition of study participants. Some studies, particularly several that did not find statistically significant intervention effects, were presented as pilot studies and were inadequately powered to detect modest effect sizes. A lack of methodologically rigorous, adequately powered studies, particularly among adolescents, makes it difficult to draw conclusions with regard to their potential for future implementation. More rigorous research in this field is critical, as is replication of studies with positive findings in other settings. Furthermore, many of the studies described in this review were multifaceted, with some delivering multiple intervention components and others providing adherence support as a part of a broader package of services; this makes it impossible to discern the relative effect of each intervention component or identify which aspects are most impactful on adherence.

Another limitation of these studies, and of adherence research as a whole, lies in the challenge of accurately measuring medication adherence and in the variety of methodologies utilized. Beyond issues of validity and precision of each measure used, it is difficult to compare the effectiveness of studies reporting different measures or different definitions of adequate adherence.

### Recommendations

Interventions that involved task shifting and community-based adherence support had the most promising evidence for adult populations, and should be tested among ALHIV. Additionally, research should examine the acceptability and cost-effectiveness of adapting **community-based adherence support** interventions for ALHIV.

Interventions that used **mHealth** platforms were numerous, but largely focused on simple adherence reminders; new strategies for using mHealth platforms to improve adherence should be developed and evaluated with a special focus on soliciting participant engagement as well as targeting the specific barriers to adherence experienced by ALHIV.

**Nutrition support** found favorable results among adults, but future implementation may be hindered by high cost and an increasingly difficult funding environment. Adaptation and testing of nutrition support interventions for ALHIV may only be warranted in settings that are amenable to long-term support for this strategy.

The two studies that evaluated the effect of **group adherence counseling** interventions for adolescents provided preliminary evidence as to its utility. Further research should be conducted on this topic, particularly focusing on providing targeted counseling for ALHIV with suboptimal adherence.

## Conclusions

We found a relatively large body of evidence on interventions to improve adherence among adults living with HIV in LMIC; however, many of these studies’ methodological quality are limited. Moreover, there is a striking lack of evidence on adherence interventions specifically for adolescents. Future research and programming should seek to answer critical questions as to whether or not existing approaches can be successfully adapted for ALHIV to address this population’s particular needs.

## Supporting information

S1 File Search strategy. (DOCX)

S2 File PRISMA checklist. (DOC)
